# Leonurine Improves Age-Dependent Impaired Angiogenesis: Possible Involvement of Mitochondrial Function and HIF-1α Dependent VEGF Activation

**DOI:** 10.3389/fphar.2017.00284

**Published:** 2017-06-06

**Authors:** Jia Qi, Jing J. Wang, Jun L. Duan, Zhao Y. Lu, Yang G. Yuan

**Affiliations:** ^1^Department of Pharmacy, Xinhua Hospital, School of Medicine, Shanghai Jiaotong UniversityShanghai, China; ^2^Department of Gerontology, Xinhua Hospital, School of Medicine, Shanghai Jiaotong UniversityShanghai, China; ^3^Department of Nephrology, School of Medicine, Shanghai Ninth People’s Hospital, Shanghai Jiaotong UniversityShanghai, China; ^4^Department of Nephrology, The First affiliated Hospital of Nanjing Medical UniversityNanjing, China

**Keywords:** leonurine, mitochondrial dysfunction, angiogenesis, hindlimb ischemia, age

## Abstract

**Objective:** Advanced age is associated with impaired angiogenesis in part because of mitochondrial dysfunction. We have recently reported that leonurine exerts protective effects in neuron via regulation of mitochondrial function. The aim of this study was to explore whether leonurine is able to attenuate mitochondrial dysfunction and to enhance angiogenesis in old rats with hindlimb ischemia.

**Methods and Results:** At day 14 after surgery, hypoxia-inducible factor (HIF)-1α and vascular endothelial growth factor (VEGF) expression was decreased in the ischemic muscle of aged animals, which was accompanied by enhanced oxidative stress, increased mitochondrial damage, decreased capillary density, and reduced limb perfusion compared with young mice. Importantly, these effects were inhibited by leonurine treatment in old animals. *In vitro*, we showed that the functional activities (migration and tube formation) of human umbilical vein endothelial cells (HUVECs) were significantly impaired in senescent compared to young. However, leonurine rescued HUVECs functional activities in senescent HUVECs. Mechanistically, we found that leonurine restored the age-dependent reduction in HIF activity and subsequent reduced VEGF expression in senescent HUVECs. Moreover, the mitochondrial oxidative stress was significantly augmented in senescent HUVECs, in association with reduced mitochondrial function. However, leonurine significantly reduced the mitochondrial oxidative stress and restored the mitochondrial membrane potential.

**Conclusion:** Our results demonstrate that leonurine protects against age-dependent impairment of angiogenesis possibly through attenuation of mitochondrial dysfunction and subsequent VEGF up-regulation impairment.

## Introduction

One of the features of aging is the decline in the ability of the organism to respond to different stresses. For example, it has been demonstrated that advanced age is associated with a defect in compensatory neovessel formation in response to tissue ischemia (Rivard et al., 1999). Moreover, angiogenic function is significantly impaired in senescent endothelial cells (ECs) (Erusalimsky, 2009). However, the mechanism of decreased angiogenesis is still elusive. Mitochondrial dysfunction is a phenomenon underlying the process of aging, which has been reported to block VEGF expression and contribute to impaired angiogenesis (Satoh et al., 2011). Importantly, mitochondria are suggested to be a site of oxygen sensing, as the electron transport chain acts as an O_2_ sensor by releasing reactive oxygen species (ROS) in response to hypoxia (Ortega-Saenz et al., 2003; Guzy and Schumacker, 2006; Klimova and Chandel, 2008). Indeed, age-related increases in oxidative damage to mitochondria are well-established in humans and model organisms (Balaban et al., 2005). Therefore, oxidative stress induced mitochondrial dysfunction may contribute to aging-associated decline in angiogenic activity.

Leonurine is an active alkaloid extracted from the traditional Chinese medicine Herba leonuri (Kong et al., 1976). It has been reported to be beneficial for cardiovascular diseases, including atherosclerosis, acute and chronic myocardial infarction (MI), and ischemic stroke (Liu et al., 2010a; Qi et al., 2010; Norata and Catapano, 2012). These beneficial effects of leonurine may be due to its various biological activities, including the preservation of mitochondrial function. Indeed, our previous studies have found that leonurine ameliorates mitochondrial dysfunction and oxidative stress in experimental stroke (Loh et al., 2010). Importantly, in rat model of chronic myocardial ischemia, leonurine was found to increase the expression of hypoxia-inducible factor (HIF)-1α and vascular endothelial growth factor (VEGF), which indicates its possible pro-angiogenic effect (Liu et al., 2010a).

Therefore, we hypothesized that leonurine may attenuate mitochondrial dysfunction and rescue age-related impairment of angiogenesis. In the present study, we investigated whether leonurine attenuates age-dependent impairment of reparative angiogenesis after ischemia, and if so, to determine the mechanism(s) involved.

## Materials and Methods

### Animals and Diet

Young male C57/BL6 male mice (4-month-old, *n* = 20) and old male C57/BL6 male mice (24-month-old, *n* = 20) were housed individually in 12 h light/dark cycles with a temperature controlled room (22 ± 2°C). All mice were allowed to have free access to standard diet and water. The research conforms to the Guide for the Care and Use of Laboratory Animals published by the US National Institutes of Health (NIH Publication No. 85-23), and the protocol was approved by the Institutional Animal Care Committee at Shanghai Jiaotong University School of Medicine.

### Generation of Ischemic Hindlimb Model

The hindlimb ischemic mouse model was generated as described previously (Lu et al., 2016). All mice were anesthetized with chloral hydrate (400 mg/kg, intraperitoneal injection), and the left femoral artery between the inguinal ligament proximally and the popliteal fossa distally was isolated and excised.

### Drug Treatments

**FIGURE 1:**
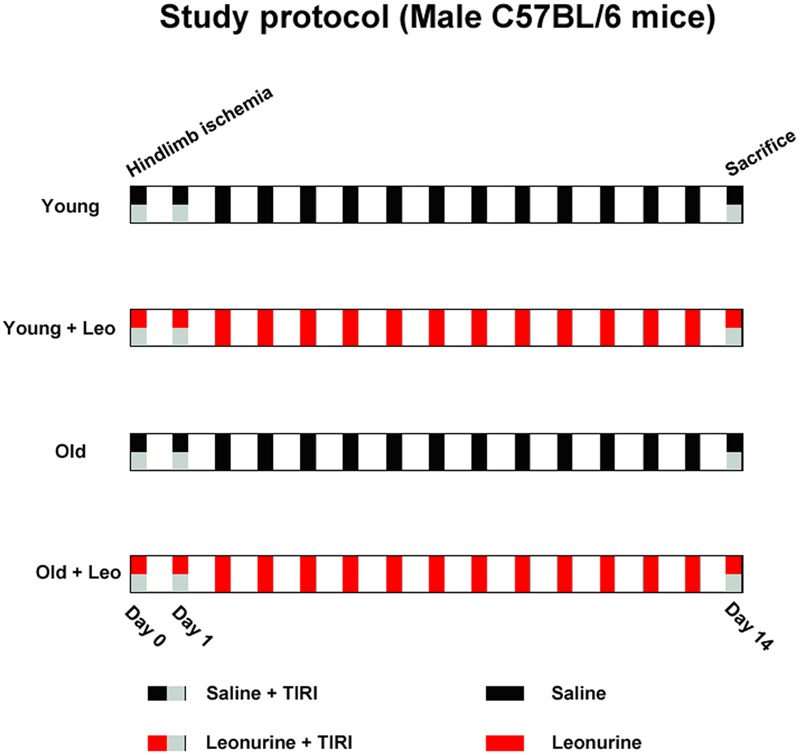
**Experimental protocol schematic**.

All mice were divided into four groups of 10 each: Young, Young + Leo (15 mg/kg/day), Old, Old + Leo (15 mg/kg/day). The dose of leonurine was based on our previous study (Liu et al., 2013). The drugs were given through intraperitoneal administration once a day. The experimental period lasts for a continuous 2 weeks (**Figure 1**).

### Necrosis Assay

The hindlimb necrosis index was performed as described previously (Bir et al., 2014). Briefly, 2-week after surgery, Gross evaluation of the mice feet were scored in terms of the following criteria: (1) there existed no eyeable necrosis; (2) minor necrosis could be captured on the nail bed; (3) necrosis were involved in all digits; (4) at least one digit was lost; and (5)two or more digits were lost or obvious necrosis.

### Thermal Infrared Imaging (TIRI) Assay

All mice skin temperatures of ischemic and non-ischemic hindlimbs were evaluated by the TIRI analyzer (Prism-DS 50137, FLIR Systems), and the color-coded images indirectly denoting changes of blood perfusion. Dark-to-purple denoted low skin temperature, and high skin temperature was showed as red-to-white colors (Lu et al., 2016). At post-operative days 0 (immediately after surgery) and 14, TIRI scans were captured, and perfusion values were obtained from histograms of colored pixels. In order to minimize the effects from surrounding light, the temperature ratio of ischemic/non-ischemic limb was employed to express blood perfusion.

### Physiology Parameters

Systolic blood pressure (SBP) and resting heart rate (HR) of mice were captured through a computerized tail-cuff system (MPA-2000, Alcott Biotech, Shanghai, China) in conscious condition. Blood glucose (GLU) data were obtained by tail-vein blood glucose measurement with OneTouch Ultra Glucometer (Johnson & Johnson, New Brunswick, NJ, United States). Moreover, body weights (BWs) were recorded before and at day 14 after leonurine administration.

### Histological and Immunofluorescence Analysis

All mice were euthanized at post-operative day 14. Ischemic and non-ischemic gastrocnemius muscles were gathered and weighed, fixed in 4% paraformaldehyde. Muscle sections (5-μm) were dyed with hematoxylin and eosin (H&E) to examine myocyte morphology. For immunofluorescence assay, tissue sections were stained using anti-CD31 antibody (BD Biosciences, Franklin Lakes, NJ, United States). 10 randomly selected fields from five independent sections in each animal (×400 magnification) were counted, and the vessel density was expressed as number of capillaries/field. To determine local oxidative stress levels, anti-nitrotyrosine antibody (Upstate, Lake Placid, NY, United States) was used. Intensities of fluorescence were measured and analyzed using Image Pro Plus software.

### Quantitative Polymerase Chain Reaction

Approximate 100 μg mid-anterior gastrocnemius fragments were homogenized in 1 ml Trizol reagent (Invitrogen, Carlsbad, CA, United States), and total mRNA was extracted. Quantitative gene expression was determined by Bio-Rad CFX96 Real-Time polymerase chain reaction (PCR) detection system using a SYBR^®^ Premix Ex Taq^TM^ Kit (Takara Bio, Inc.). Each targeted mRNA expression was normalized to its glyceraldehyde 3-phosphate dehydrogenase (GAPDH) quantity. The primers used as follows:

Human VEGF

Forward: 5′-GGGCAGAATCATCACGAAGT-3′;
Reverse: 5′-GGCTTGAAGATGTACTCGATCTC-3′

Human GAPDH

Forward: 5′-GGGAAACTGTGGCGTGAT-3′;
Reverse: 5′-AAAGGTGGAGGAGTGGGT-3′

Mouse ND1

Forward: 5′-CTCAACCTAGCAGAAACAAACC-3′;
Reverse: 5′-GGCCGGCTGCGTATTCTAC-3′

Mouse cytochrome b

Forward: 5′-AAAGCCACCTTGACCCGATT-3′;
Reverse: 5′-GATTCGTAGGGCCGCGATA-3′

Mouse GAPDH

Forward: 5′-CTCAACTACATGGTCTACATGTTCCA-3′;
Reverse: 5′- CCATTCTCGGCCTTGACTGT-3′.

### Mitochondrial DNA Short-and Long-Extension PCR Amplification

The mtDNA was isolated with a mitochondrial DNA kit (Biovision, United States) according to the manufacturer’s protocol. Long and short mtDNA fragments were co-amplified by Long-range PCR (Mansouri et al., 1999).

### Cell Culture

Human umbilical vein endothelial cells (HUVECs) (ATCC, Cat. CRL1730) were maintained in Dulbecco’s Modified Eagle Medium (DMEM) at 37°C in 95% air and 5% CO_2_. Passaging was always performed at 80–90% confluence, and HUVECs were serially passaged until the growth retard state. Passage 4–5 HUVECs were termed as “young cells” and Passage 21–22 as “senescent cells” as described previously (Hoffmann et al., 2001). Senescence-associated β-galactosidase activity was analyzed as described previously (Dimri et al., 1995). HUVECs were grouped into four experimental groups: Young, Young + Leo (10 μmol/L), Senescent, Senescent + Leo (10 μmol/L). HUVECs were stimulated with hypoxia to mimic *in vivo* ischemia as described previously (Han et al., 2016). All experimental groups were maintained in a hypoxic chamber (37°C, with 94% N_2_, 1% O_2_, and 5% CO_2_) (Thermo Fisher Scientific, Waltham, MA, United States) for different periods, and leonurine was added 30 min before hypoxia. Cell migration was determined by Boyden Transwell chambers (Corning, Cambridge, MA, United States) assay. Tubule formation was evaluated by Matrigel (BD Biosciences, Bedford, MA, United States) assay. Mitochondrial superoxide was quantitatively computed by using MitoSOX Red mitochondrial superoxide indicator (Invitrogen, Carlsbad, CA, United States) as the manufacturer recommended. Mitochondrial membrane potential was determined by using JC-1 (Invitrogen, Carlsbad, CA, United States). The experiments were repeated three times.

### Galactosidase Staining

Senescence-associated β-galactosidase (SA-β-gal) activity was performed according to the manufacturer’s instructions (Beyotime, Haimen, China). The percentage of SA-β-gal positive cells was evaluated by light microscopy (×100 magnification).

### Tubule Formation Assay

Briefly, 96-well dishes were coated with Matrigel (BD Biosciences, Bedford, MA, United States), HUVECs (2.5 × 10^4^/well) were seeded and incubated with or without leonurie for 30 min. Thereafter, HUVECs were incubated for 6 h in hypoxic chamber, and graphs were captured with a light microscopy (×100 magnification). Tubule length was expressed as fold of control.

### Migration Assay

Migration assay was carried out using a 24-well Boyden chambers (Corning, Cambridge, MA, United States). In brief, medium (600 μL) with or without leonurine were added to the lower chambers, HUVECs (2 × 10^4^/well/100 μL) were added to the top wells in serum-free Medium for 30 min. Thereafter, HUVECs were incubated for 6 h in hypoxic chamber. The HUVECs that migrated to the bottom of membranes were fixed and stained with 0.1% crystal violet, and migrated cells were counted in six randomly chosen fields in three independent experiments with light microscopy (×100 magnification).

### ChIP Assay

ChIP assay was employed in HUVECs as described previously (Wang et al., 2008). The region from -1140 to -788 of the VEGF promoter was amplified by PCR from immunoprecipitated chromatin with the following primers:

Forward: 5′-GCGGGTAGGTTTGAATCATC-3′;
Reverse: 5′-GC CTGCAGACATCAAAGTGA-3′.

### Western Blot Analysis

Equal amounts of Protein extracts from mid-anterior gastrocnemius and HUVECs were subjected to SDS-PAGE, electrotransfer, and then blotted with anti- HIF-1α (Santa Cruz Biotechnology, United States), anti-VEGF, anti-p21 (Abcam, UK) and anti-GAPDH (Beyotime, Haimen, China). Then, the membranes were incubated with IRDye800CW conjugated secondary antibody. Targeted bands were detected by Odyssey imaging system (LICOR).

### Measurement of Mitochondrial Oxidative Stress and Membrane Potential

To assess mitochondrial superoxide, cells were dyed with MitoSOX for 10 min before fixed with 4% paraformaldehyde. The cells were analyzed under fluorescence confocal microscopy and fluorescence intensity was measured in 4–5 independent fields. For measurement of the mitochondrial membrane potential, cells were incubated with 200 nM JC-1 for 30 min before analyzed under fluorescence confocal microscopy. The ratio of red and green fluorescence was used to evaluate mitochondrial membrane potential.

### Statistical Analysis

Values were represented as mean ± SEM. Two-way ANOVA was performed to determine the age and condition factors. Statistical significance was analyzed by one-way analysis of variance (ANOVA) followed by Tukey’s *post hoc* test. A value of *p* < 0.05 was accepted statistical significance.

## Results

### Effects of Aging and Leonurine on Hemodynamic and Physical Characteristics

Hemodynamic data and physical characteristics of the animals before surgery and on day 14 after limb ischemia are shown in **Table 1**. 14 days after treatment, statistical differences of basal HR, SBP and random blood glucose among four groups were not observed. Although BW was significantly affected by age, statistically significant differences were not captured in Young + Leo and Old + Leo groups relative to their untreated groups.

**Table 1:** Effect of leonurine on physiological parameters in young and old mice.

	Young	Young + Leo	Old	Old + Leo
SBP(mmHg)				
Before surgery	121.73 ± 5.57	121.30 ± 5.21	131.37 ± 3.14	130.58 ± 3.75
14 Day	121.44 ± 4.10	122.91 ± 5.35	131.08 ± 4.73	132.06 ± 3.41
Weight(g)				
Before surgery	26.83 ± 1.19	26.00 ± 1.24	35.00 ± 1.41^∗∗^	35.5 ± 0.99
14 Day	26.17 ± 0.98	26.67 ± 1.15	35.67 ± 0.88^∗∗^	35.17 ± 1.14
HR(beats/min)				
Before surgery	356.00 ± 10.21	352.00 ± 8.43	345.67 ± 8.07	347.50 ± 8.15
14 Day	352.83 ± 9.57	348.17 ± 7.29	361.33 ± 6.96	354.16 ± 4.33
GLU(mmol/L)				
Before surgery	5.83 ± 0.21	5.30 ± 0.23	5.52 ± 0.14	5.33 ± 0.13
14 Day	5.47 ± 0.16	5.60 ± 0.36	5.75 ± 0.17	5.40 ± 0.23

*^∗∗^*p* < 0.01 vs. Young.*

### Leonurine Improves Blood Flow Recuperation in Old Ischemic Muscles

**FIGURE 2:**
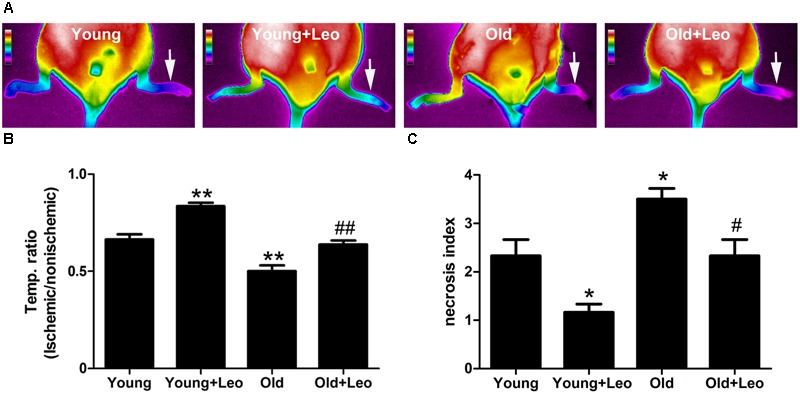
**Effects of aging and leonurine on blood flow recuperation.** Hindlimb perfusion was measured with TIRI analyzer and expressed by the ischemic/non-ischemic limb temperature ratios. **(A)** Representative results of TIRI measurements 14 days after hindlimb ischemia operation in different groups of mice. A color scale represents blood flow variations from minimal (dark blue) to maximal (white) values. Arrows indicate ischemic (left) hindlimb. **(B)** Quantification of Reperfusion values represented by changes in ischemic/non-ischemic limb perfusion. **(C)** Necrosis scores evaluated on day 14. Values are expressed as mean ± SEM. ^∗^*p* < 0.05 vs. young group; ^∗∗^*p* < 0.01 vs. young group; ^#^*p* < 0.05 vs. old group; ^##^*p* < 0.01 vs. old group. *n* = 10 for each group.

To investigate the role of leonurine on the age-dependent modulation of neovascularization, hindlimb ischemia was surgically induced in mice. TIRI was then used to evaluate the hindlimb perfusion. Aging was associated with a significant impairment of blood flow recuperation at day 14 (Old 0.50 ± 0.03 vs. Young 0.66 ± 0.03; *p* < 0.05) after surgery. However, blood perfusion was significantly higher in Young + Leo (0.84 ± 0.02) when compared with Young and leonurine remarkably augmented blood flow in Old + Leo (0.64 ± 0.02), bringing them up to the values of Young (**Figures 2A,B**). To study the effect of leonurine on tissue viability, we assessed the necrosis scores of the ischemic limbs 14 days after surgery. We found that the ischemic scores of leonurine-treated mice were significantly lower than that in non-treated ones either between young groups or old groups, which indicates that leonurine is effective in recovering blood perfusion and preserving tissue viability after ischemia (**Figure 2C**).

### Leonurine Increases Muscle HIF-1α and VEGF Expression and Promotes Angiogenesis after Hindlimb Ischemia

**FIGURE 3:**
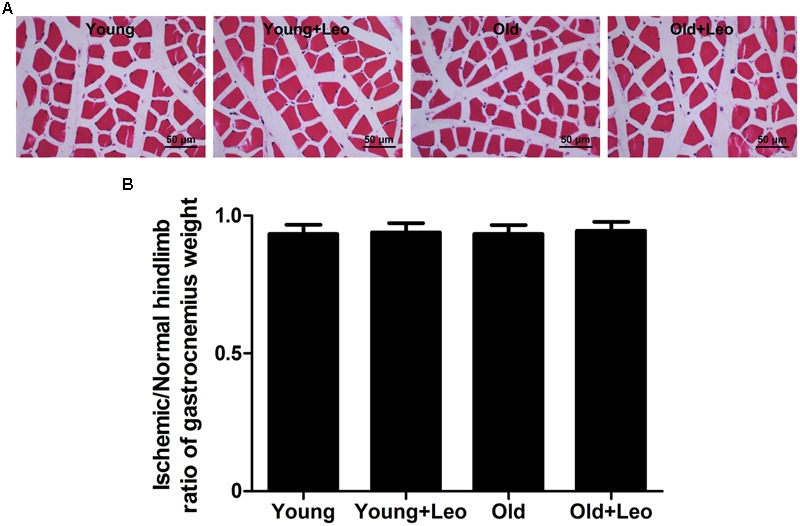
**H&E-staining of ischemic hindlimb muscles of different groups. (A)** Representative H&E-staining images. Scale bar = 50 μm. **(B)** Weight of left (ischemic) gastrocnemius muscle normalized to right.

**FIGURE 4:**
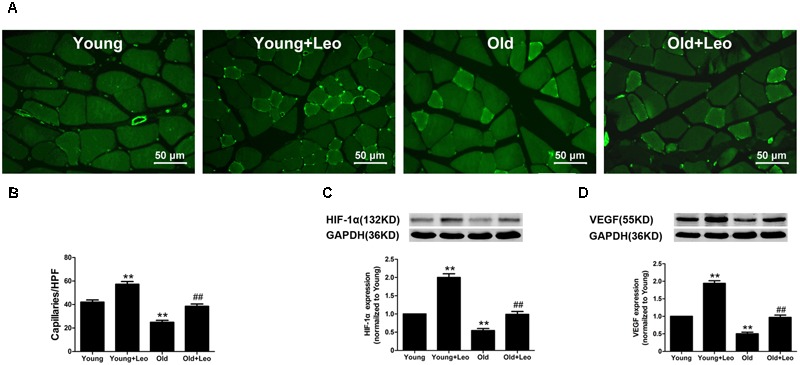
**Effects of aging and leonurine on capillary density and expressions of HIF and VEGF. (A)** CD31 immunostaining of ischemic skeletal muscles harvested at post-surgical day 14 in each group. Scale bar = 50 μm. **(B)** Quantification of capillary density. **(C,D)** Effects of aging and leonurine on the expressions of HIF and VEGF in ischemic skeletal muscles assessed by Western blot. Data are mean ± SEM. ^∗∗^*p* < 0.01 vs. young group; ^##^*p* < 0.01 vs. old group. *n* = 5–8 for each group.

To determine the changes of myocyte morphology in respond to ischemic injury, H&E sections were performed. The results indicated approximately normal myocyte morphology, with obvious muscle cells rounding, remarkably centralized nuclei, significant inflammatory cell or adipocyte were not emerged in all four groups (**Figure 3A**). Meantime, there existed no evident difference in the weight ratio of the ischemic/non-ischemic gastrocnemius muscle among the four groups (**Figure 3B**). Anti-CD31 staining was performed in all animals at day 14 after surgery to determine the capillary density. It showed that aging was associated with a significant reduction of capillary density in ischemic muscles. For capillary rarefaction was recorded in old mice relative to young mice. However, leonurine was efficient in increasing the number of capillaries by 36% in Young + Leo and restoring it in Old + Leo (**Figures 4A,B**). To elucidate the underlying molecular mechanisms of leonurine-induced angiogenesis, we subsequently measured the expressions of VEGF and HIF-1α in skeletal muscles at day 14 after surgery. When compared with Young group, leonurine increased all these protein expressions in Young + Leo group (**Figures 4C,D**). As expected, angiogenic factors were evidently retarded in Old compared with Young. Interestingly, this inhibition can be rectified by leonurine (**Figures 4C,D**), which indicated that VEGF and HIF-1α could act synergistically for appropriate vascular growth.

### Leonurine Reduces Oxidative Stress Levels in Old Ischemic Tissues

**FIGURE 5:**
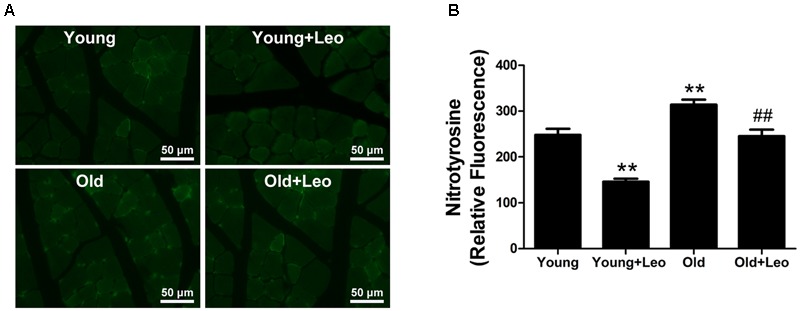
**Effects of leonurine on oxidative stress levels in ischemic muscles. (A)** Representative photomicrographs of nitrotyrosine immunostaining in ischemic muscles harvested at day 14 after ischemia in the different groups. **(B)** Quantification of nitrotyrosine immunostaining in the ischemic muscles of mice from the different groups. Data are mean ± SEM. ^∗∗^*p* < 0.01 vs. young group; ^##^*p* < 0.01 vs. old group. *n* = 6–8/group.

To determine the oxidative stress level in ischemic tissues, we performed immunostaining for the oxidative stress marker nitrotyrosine. Nitrotyrosine immunostaining demonstrated that oxidative stress level was significantly increased in ischemic muscles of old mice compared with young group. However, leonurine attenuated the level of oxidative stress in Young + Leo and normalized age-dependent increase of oxidative stress levels in Old + Leo when compared with Young group (**Figures 5A,B**).

### Leonurine Attenuates Mitochondrial Damage in Old Ischemic Muscles

**FIGURE 6:**
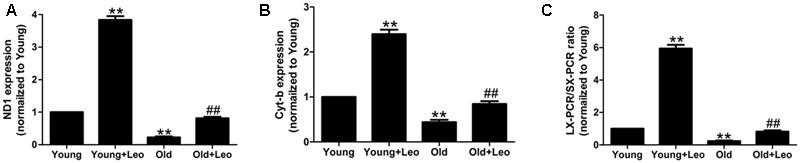
**Effects of aging and leonurine on mitochondrial damage in ischemic muscles. (A–C)** Quantification of mRNA expression of mitochondrial component, nicotinamide adenine dinucleotide dehydrogenase subunit 1 (ND1; **A**) and cytochrome b (Cyt-b; **B**) assessed by quantitative realtime PCR. **(C)** Quantification of Long-extension (LX)-PCR and short-extension (SX)-PCR of mtDNA ratio. Data are mean ± SEM. ^∗∗^*p* < 0.01 vs. young group; ^##^*p* < 0.01 vs. old group. *n* = 6–8/group.

Mitochondrial damage is one of the most widely recognized causes of aging. Our previous studies have reported the protective effects of leonurine on mitochondria in neuron. To investigate whether the anti-oxidative effect of leonurine is involved in its pro-angiogenic effect in old mice, we examined mRNA expression of nicotinamide adenine dinucleotide dehydrogenase subunit 1, and cytochrome b. Both of these genes were decreased in old compared with young rats, which were restored by leonurine. In addition, leonurine augmented these gene levels in Young + Leo relative to control levels (**Figures 6A,B**). Long-extension PCR and short-extension PCR of mtDNA were performed to determine the effect of leonurine on deleted mtDNA molecules in ischemic muscles. Deletions of mtDNA in muscles were found to be associated with aging, which were attenuated by leonurine (**Figure 6C**).

### Leonurine Increases VEGF Expression and Secretion and Restores Endothelial Function in Senescent HUVECs

**FIGURE 7:**
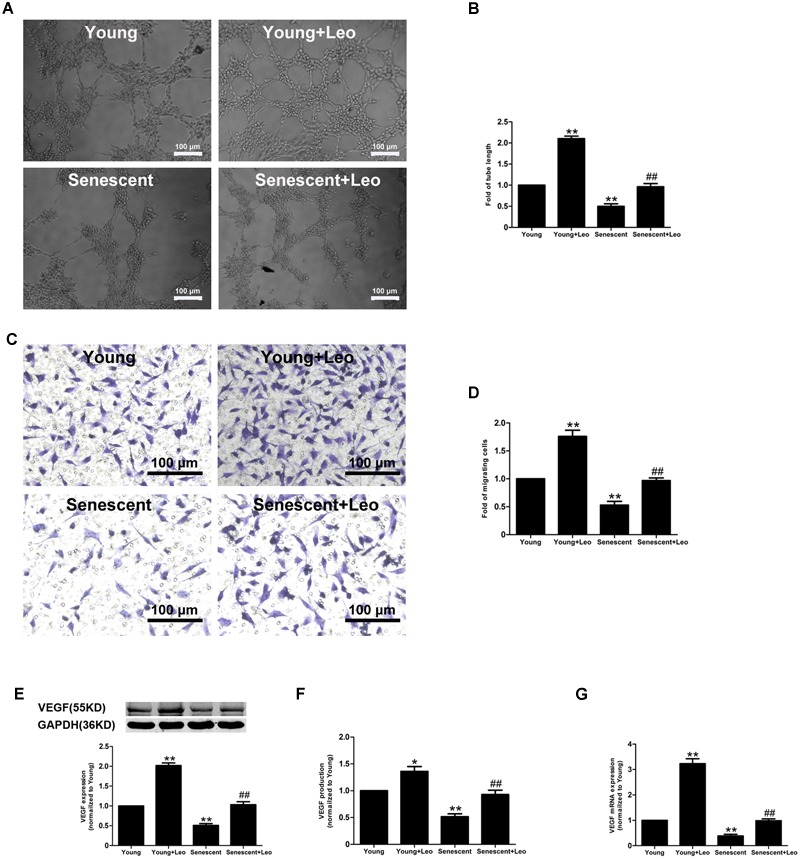
**Effects of leonurine on the endothelial functions in senescent HUVECs. (A,B)** Representative micrographs and quantification of tube formation in HUVECs treated as indicated (*n* = 3 each). Scale bar = 100 μm. **(C,D)** Representative images and quantitative analysis of cell migration with migration assay in HUVECs treated as in **(A)** (*n* = 3 each). Scale bar = 100 μm. **(E–G)** Effects of aging and leonurine on VEGF expression, secretion and mRNA expression in HUVECs. ^∗^*p* < 0.05 vs. young group; ^∗∗^*p* < 0.01 vs. young group; ^##^*p* < 0.01 vs. senescent group. Data are mean ± SEM.

Cellular senescence in ECs has been evidenced to lead to significantly attenuated angiogenic functions. To investigate the impact of leonurine on senescent endothelial angiogenic functions, we performed the senescent HUVECs *in vitro*, and passage 21–22 HUVECs exhibited much more SA-β-gal activity (**Supplementary Figure S1**). Next, we examined the angiogenic capacities of HUVECs. ECs migration and tube formation on matrigel in response to hypoxia were significantly attenuated in senescent ECs as compared with those in young cells. However, both ECs migration and tube formation in response to hypoxia were significantly enhanced by the treatment of leonurine in both young and senescent HUVECs (**Figure 7**). As migration and proliferation of ECs in response to VEGF play an important role in angiogenesis, we also investigated the effect of senescence and leonurine on VEGF expression and secretion in ECs. VEGF protein expression and secretion showed a statistically significant decrease in senescent HUVECs, whereas leonurine increased VEGF protein expression and secretion both in young and senescent HUVECs (**Figure 7**). These results strongly suggest that leonurine may preserve EC functions in senescent HUVECs, which may involve the restoration of VEGF expression.

### Leonurine Increases VEGF Expression in Senescent HUVECs via Activation of HIF-1α

**FIGURE 8:**
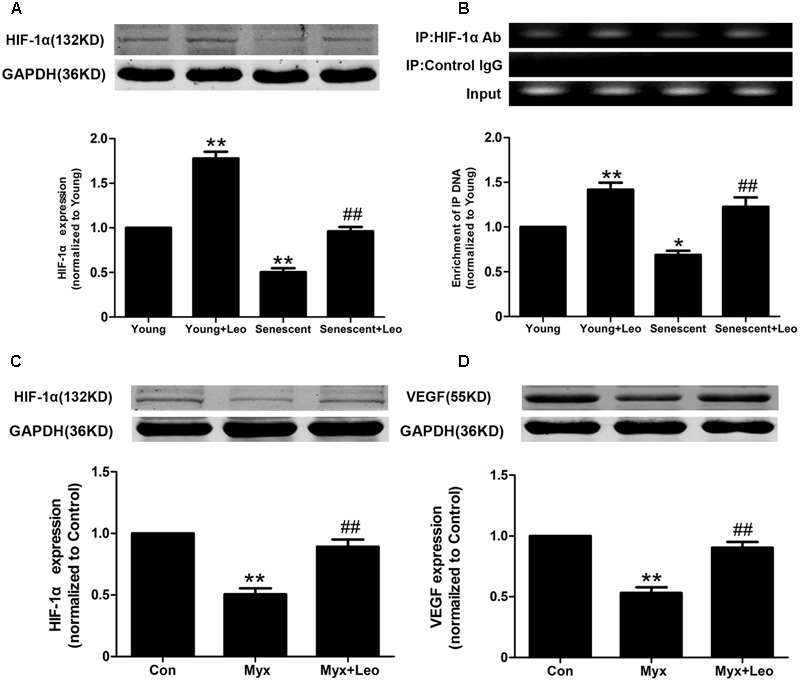
**Leonurine increases VEGF expression in senescent HUVECs via activation of HIF-1α. (A)** The effects of aging and leonurine on HIF-1α expression in HUVECs detected by Western-blot. **(B)** The effects of aging and leonurine on the binding of HIF-1α to VEGF gene promoters detected by chromatin immunoprecipitation (ChIP). **(C,D)** Leonuine restores the expresssions of HIF and VEGF compromised by myxothiazol, an inhibitor of mitochondrial complex III. ^∗^*p* < 0.05 vs. young group; ^∗∗^*p* < 0.01 vs. young group; ^##^*p* < 0.01 vs. senescent group. Data are mean ± SEM.

HIF-1α is one of key transcriptional regulators of VEGF gene. It has been reported that age-dependent reduction of hypoxia-induced VEGF expression is due to reduced HIF-1 activity and may explain the age-dependent impairment of angiogenesis in response to ischemia. Therefore, we assessed the protein expression and activation of HIF-1α by western-blot and ChIP assay respectively. As expected, HIF-1α expression under hypoxic condition was reduced in senescent HUVECs compared with Young, which was restored by leonurine treatment. In addition, leonurine enhanced the protein level in Young + Leo in comparison with control levels (**Figure 8A**). In accordance with previous studies, the HIF-1 activity under hypoxic condition was decreased in senescent HUVECs compared with Young. However, leonurine enhanced the HIF-1α activation in both young and senescent HUVECs (**Figure 8B**). Mitochondrial dysfunction and impaired HIF-1α activation in response to hypoxia induced by myxothiazol were also improved by leonurine (**Figures 8C,D**).

### Leonurine Reduces the Mitochondrial Oxidative Stress and Restores the Mitochondrial Functions in Senescent HUVECs

**FIGURE 9:**
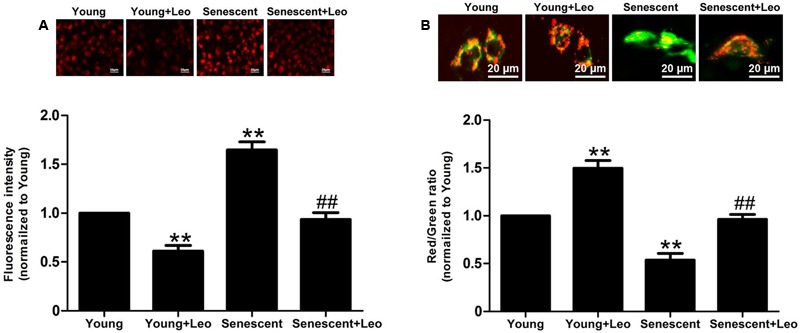
**Leonurine reduces the mitochondrial oxidative stress and restores the mitochondrial functions in senescent HUVECs. (A**) The effects of aging and leonurine on mitochondrial superoxide detected by MitoSOX. **(B)** The effects of aging and leonurine on mitochondrial membrane potential assessed by the red/green ratio of JC-1 staining. ^∗∗^*p* < 0.01 vs. young group; ^##^*p* < 0.01 vs. senescent group. Data are mean ± SEM.

It has been reported that mitochondria were essential for VEGF expression through HIF-1α activation in response to hypoxia and the accumulation of mitochondrial damage may cause senescence. So we analyzed the mitochondrial oxidative stress by using MitoSOX and found the augmented mitochondrial oxidative stress in senescent HUVECs. Moreover, JC-1 staining showed significantly attenuated mitochondrial membrane potential in senescent HUVECs. However, of note, leonurine reduced the mitochondrial superoxide and restored the membrane potential in senescent HUVECs. In addition, the protective effect of leonurine also emerged in Young + Leo in comparison with control levels (**Figure 9**). The results indicate that leonurine may attenuate the impaired angiogenic functions in senescent ECs partly by inhibiting the mitochondrial oxidative stress.

## Discussion

The present study was to determine the effect of leonurine on age-dependent impairment of angiogenesis in response to tissue ischemia. In old rats, leonurine promotes vascular growth in the ischemic limb, improves blood perfusion, and preserves tissue viability. In addition, the increase of HIF-1α and VEGF expression in old ischemic muscles mediates the positive effects of leonurine on angiogenesis. Our data further demonstrate that leonurine mitigates mitochondrial dysfunction and restores HIF-1-dependent VEGF up-regulation in senescent HUVECs.

Age-dependent impairment of angiogenesis may account for the increased severity of cardiovascular diseases in the geriatric population (Leosco et al., 2007). In old animals with critical limb ischemia, limb perfusion, and muscle capillarization are significantly reduced compared with younger animals (Rivard et al., 1999). The present study is in agreement with previous observations showing that age-related compromise of the compensatory neovessel formation represents, at least in part, a reversible phenomenon. Indeed, as shown here, leonurine improved new vessel development, blood perfusion, and tissue viability of ischemic limbs of old animals. In addition, leonurine showed a greater effect on old animals, which may due to the more evident positive effect of leonurine on responses to ischemia in impaired angiogenesis rather than preserved, such as the younger ones.

It has been shown that age-dependent impairment of angiogenesis primarily results from a defect in the transcriptional regulation of VEGF (Rivard et al., 1999). More recently, it has been reported that age-dependent alterations of transcriptional and post-transcriptional regulation of HIF-1 are key determinants of VEGF dysregulation in aged (Rivard et al., 2000). HIF-1 is specifically involved in the regulation of muscle adaptations after hypoxia. Our previous study has found that leonurine increases HIF-1α and VEGF expression in rats in chronic infarction (Liu et al., 2010a). Our present data is in agreement with the previous results and demonstrates for the first time that leonurine is able to restore HIF-1α and VEGF expression both in senescent HUVECs and old ischemic muscles. It is likely that the overall improvement of age-impaired may improve restoration of VEGF expression in response to ischemia.

Mitochondria are essential for VEGF expression via HIF-1α activation in response to hypoxia, which aligns with the important role of mitochondrial ROS for hypoxic activation of HIF-1 (Bell et al., 2007; Satoh et al., 2011). Moreover, mitochondrial function inhibition reverses hypoxia-induced HIF-1 activation, which leads to the speculation that mitochondria play a role in cellular oxygen sensing. Indeed, inhibition of mitochondrial complex III by antimycin A1 inhibits HIF-1 activation and angiogenesis by decreasing VEGF expression (Maeda et al., 2006). Of note, a recent study reported that HIF-1 activation in response to hypoxia was abolished in Mitochondrial DNA depleted human proximal tubular epithelial cells (hPTECs) and in myxothiazol-treated hPTECs (Satoh et al., 2011). Thus, angiogenic capacity may worsen as a result of mitochondrial dysfunction in old animals. Our previous studies have demonstrated that leonurine is able to attenuate mitochondrial dysfunction in neuron and cardiomyocytes (Liu et al., 2010b; Loh et al., 2010). Here, we found that leonurine may restore the mitochondrial function in senescent HUVECs and old ischemic muscles and reverse hypoxic activation of HIF compromised in senescent HUVECs and in myxothiazol-treated HUVECs.

Reactive oxygen species of the mitochondrial respiratory chain is important for activation of HIF-1 in hypoxia, which participates in signal transduction pathways involved in angiogenesis. However, ROS overplus induced by damaged mitochondria prevents the assembly of superoxide-generating enzyme complexes. In fact, damaged mitochondria show low membrane potential and low ATP production, along with which ROS production, dependent on a normal proton concentration gradient, is low. In addition, it has been reported that mitochondria-targeted antioxidant SkQ1 stimulated angiogenesis and improved wound healing in old mice. We have previously reported that leonurine inhibited mitochondrial ROS production and regulated mitochondrial function in neuron. The present study demonstrates for the first time that leonurine was able to inhibit mitochondrial ROS production augmented in senescent ECs. However, augmenting the gene expression of mitochondrial genes by leonurine indicate that it could have direct roles in inducing gene expression. The anti-oxidative property of leonurine could be a parallel effect on hypoxia being an antioxidant. Further studies are needed to clarify it.

## Conclusion

This is the first demonstration of therapeutic potential of leonurine on age-dependent impairment of angiogenesis. The present study revealed that leonurine improves endothelial angiogenic functions impaired by cellular senescence in ECs, at least partly involved in inhibiting mitochondrial dysfunction and restoring HIF-1-dependent VEGF upregulation in senescent HUVECs. Meanwhile, administration of leonurine improves new vessel development, blood perfusion, and viability of ischemic limbs of old animals associated with activation of a HIF-VEGF pathway. The pro-angiogenic mechanism by which leonurine regulates various signaling pathways is still undetermined and needs to be clarified in the near future.

## Author Contributions

JQ, JW, and JD designed the research work; ZL, YY, and JQ performed the research experiments; JW contributed new reagents/analytic tools; JQ analyzed data; YY and JQ wrote the paper.

## Conflict of Interest Statement

The authors declare that the research was conducted in the absence of any commercial or financial relationships that could be construed as a potential conflict of interest. The reviewers J-LH, WL and handling Editor declared their shared affiliation, and the handling Editor states that the process nevertheless met the standards of a fair and objective review.

## Supplementary Material

FIGURE S1 **Leonurine decreased cell senescence in HUVECs. (A)** SA-β-gal staining in young and senescent HUVECs. **(B)** The effects of aging and leonurine on p21 expression.
