# Supplementary figures and images for: Leonurine Improves Age-Dependent Impaired Angiogenesis: Possible Involvement of Mitochondrial Function and HIF-1α Dependent VEGF Activation
Source: Front Pharmacol. 2017 Jun 6;8:284. doi: 10.3389/fphar.2017.00284 (PMC5459903; doi:10.3389/fphar.2017.00284)

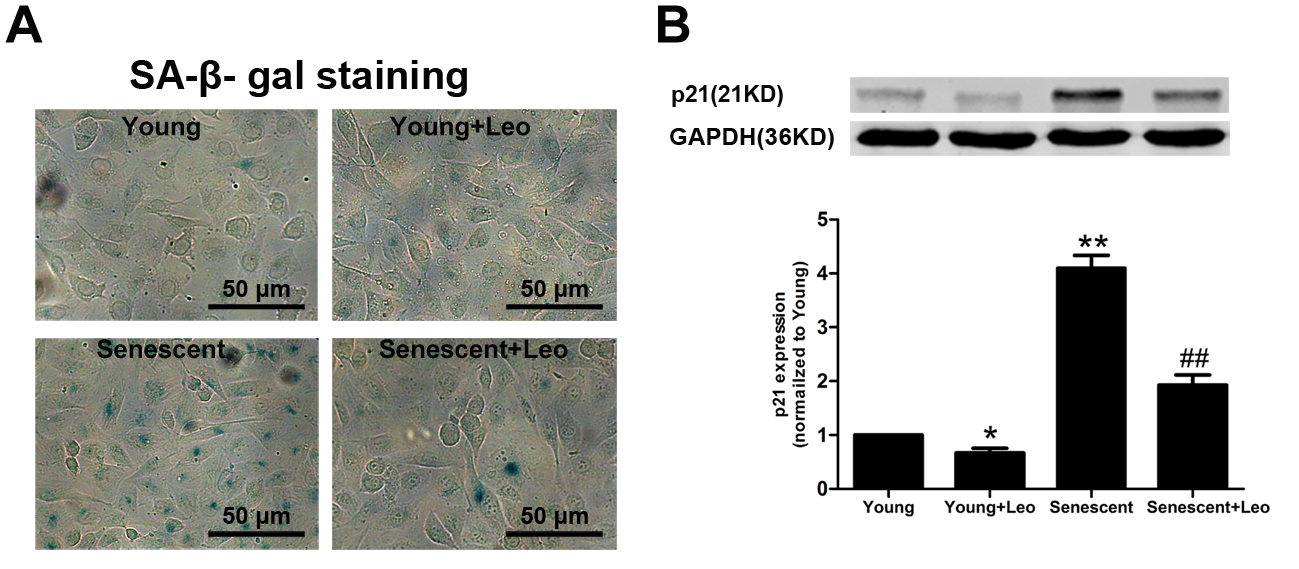

Supplement: FIGURE S1 — Leonurine decreased cell senescence in HUVECs. (A) SA-β-gal staining in young and senescent HUVECs. (B) The effects of aging and leonurine on p21 expression. [file Image_1.TIF]
